# An inhomogeneous across-slab conduit controlled by intraslab stress heterogeneity in the Nankai subduction zone

**DOI:** 10.1038/s41598-018-38142-w

**Published:** 2019-01-30

**Authors:** Makoto Otsubo, Ayumu Miyakawa, Ikuo Katayama, Keishi Okazaki

**Affiliations:** 1 Geological Survey of Japan, AIST, Tsukuba Central 7, 1-1-1 Higashi, Tsukuba, 305-8567 Japan; 2 Department of Earth and Planetary Systems Science, Hiroshima University, Higashi-Hiroshima, 739-8526 Japan; 3 Kochi institute for Core Sample Research, Japan Agency for Marine-Earth Science and Technology, 200 Monobe Otsu, Nankoku, 783-8502 Japan

## Abstract

Nonvolcanic, deep low-frequency tremors and slow-slip events occur simultaneously in the transition zone from locked to continuously creeping fault in the down-dip portion of the Nankai Trough subduction zone, southwestern Japan. The occurrence of these slow earthquakes is discontinuous along the trench and attributed to the effect of high pore pressures at the plate boundary. Here, we show that spatial variations in intraslab stress may control fluid migration from the subducted Philippine Sea slab to the plate boundary. The triaxial normal faulting stress, detected by stress tensor inversion using focal mechanisms in the slab, controls anisotropically permeability that trends NNW–SSE subhorizontally from the subducted Philippine Sea slab to the plate boundary. The inhomogeneous permeability controlled by spatial stress heterogeneities in the subducted Philippine Sea slab controls the intraslab fluid pathway. This hypothesis is consistent with the spatial heterogeneity of slow earthquakes and ^3^He/^4^He ratio distributions.

## Introduction

**Figure 1:**
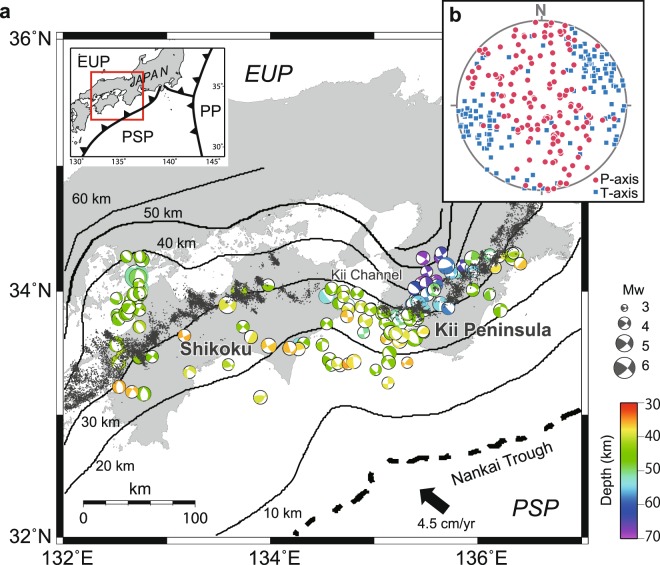
Seismicity in the Philippine Sea slab beneath the Shikoku district and the Kii Peninsula. (**a**) Focal mechanism solutions in the Philippine Sea slab beneath the Shikoku district and the Kii Peninsula; the colour of focal spheres indicates hypocentre depths. (**b**) Red circles and blue squares indicate P- and T-axes of the solutions, respectively. In a, grey dots indicate epicentres of nonvolcanic tremors in southwestern Japan for the period 2001 to 2009^32^, depth contours indicate the upper surface of the Philippine Sea plate^33^, and arrow indicates the convergence vector between the Philippine Sea Plate (PSP) and the Japanese Islands (Eurasian Plate, EUP)^34^. PP, Pacific Plate.

High pore fluid pressure in subduction zones has been recognized as playing an important role in the occurrence of nonvolcanic, deep low-frequency tremors and slow-slip events, causing a reduction in frictional strength and fault instability at the plate interface^1^. Water in the subducting plate is released into the overlying mantle wedge by the dehydration of hydrous minerals in subducting oceanic crust^2^. At some island arcs, a contrast in permeability across the Moho results in the accumulation of water and the build-up of high pore fluid pressure in the corner of the mantle wedge overlying the subducting plate^3^. A discontinuous band of slow earthquakes along the Nankai subduction zone is observed in the Kii channel, which is located between the Kii Peninsula and the Shikoku district in Japan (Fig. 1)^4^. The spatial distribution of hydrous mineral dehydration and consequent build-up of high pore fluid pressure in the corner are nearly homogeneous along the Nankai subduction zone^5^, implying that the discontinuous occurrence of slow earthquakes are not controlled by the dehydration. The discontinuous band may be caused by spatial heterogeneities in the state of stress in the Philippine Sea slab beneath the mantle wedge.

Under the stress and temperature conditions of a subducted slab, fluids are able to migrate along permeable fractures. Spatial variations in stresses generate faults and shear fractures, which may act as conduits for hydromigration and/or increase the permeability of the slab. Such stresses also control the opening and closing of existing fractures^6^. In this study, we examine the hypothesis that spatial stress variation controls intraslab fluid migration by calculating the heterogeneity in intraslab stresses, on the basis of which we propose a fluid pathway from the slab to the corner of mantle wedge where the fluid pressures facilitate the occurrence of slow earthquakes.

## Seismic events in the Philippine Sea slab

We used focal mechanism data of seismic events in the Philippine Sea slab beneath the Shikoku district and the Kii Peninsula to infer the present-day intraslab stresses (Fig. 1). Recent studies have revealed that the state of stress is spatially heterogeneous at a regional scale^7^, and earthquake focal mechanisms may also be heterogeneous. Numerical techniques are essential for detecting the particular heterogeneity in the stress state. We used the multiple inverse method (MIM)^7^ to separate stresses derived from earthquake focal mechanism data from the spatially variable state of stress.

Figure 1 shows focal mechanism data of seismic events in the Philippine Sea slab beneath Shikoku and the Kii Peninsula, which are made publicly available by the National Research Institute for Earth Science and Disaster Prevention (NIED), Japan. The data correspond to 188 seismic events that occurred between 1 January 1997 and 31 December 2010. All the foci were located in the depth range of 35–70 km, and all the events had magnitudes exceeding 3.2 (Fig. 1a). The P- and T-axes of the focal mechanisms are illustrated in Fig. 1b. Most of the events represent normal and strike-slip faulting with NE–SW- to E–W-trending T-axes (Fig. 1b).

### Separating stresses from heterogeneous focal mechanism data

**Figure 2:**
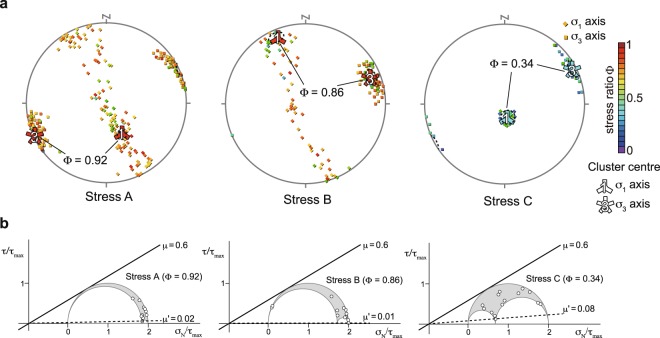
Crustal stresses in the Philippine Sea slab beneath the Shikoku district and the Kii Peninsula. (**a**) Lower-hemisphere equal-area stereographic projections showing the results of the multiple inverse method^7^ applied to data from the 188-point dataset of Fig. 1, and the results of the k-means clustering^31^ applied to the data. Diamonds and squares indicate σ_1_- and σ_3_-axes, respectively. The colour of the symbols represents the stress ratio. Dashed circles show 95% confidence intervals of the stresses. (**b**) Analysis of dimensionless Mohr diagrams obtained for the Philippine Sea slab beneath the Shikoku district and the Kii Peninsula. The lower boundary of the mass of representative points is shown as a broken line. We classified 36 earthquake events into three groups as F1, F2, and F3 to estimate the effective friction coefficient *μ*′ for each fault group compatible only with the stress. We used a value of 0.6 for the friction coefficient *μ*^16^.

Three stresses, labelled A, B, and C, were detected by the MIM (Fig. 2a). Stress A has σ_1_ and σ_3_ orientations (strike/dip) of 153°/55° and 245°/2°, respectively, and a stress ratio Φ (Φ = (σ_2_ - σ_3_)/(σ_1_ - σ_3_)) of 0.92 (Fig. 2a) where σ_1_, σ_2_, and σ_3_ are the maximum, intermediate, and minimum principal stress magnitudes, respectively. The second solution, Stress B, has σ_1_ and σ_3_ orientations of 337°/13° and 70°/12°, respectively, and a stress ratio Φ of 0.86 (Fig. 2a). The third solution, Stress C, has σ_1_ and σ_3_ orientations of 170°/76° and 62°/4°, respectively, with a stress ratio Φ of 0.34 (Fig. 2a). The σ_1_ and σ_3_ orientations of the stresses do not overlap at 95% confidence intervals of the stresses. To judge a stress as fitting a datum, the stress must explain slip vectors on either nodal plane for all 188 events. When a stress with an angular misfit of <30° is judged to be compatible with a fault-slip datum^8^, either or both of the stresses explain slip vectors on one of the nodal planes for 164 of the 188 events. The thresholds of the angular misfit were determined here based on the uncertainties of the strike, dip, and rake^9,10^. When the angular misfits are smaller than the uncertainties of the focal mechanisms, the observed slip directions agree with theoretical directions to within the estimated the uncertainties. Of the 164 events, only 36 are compatible only with a single principal stress, as the stress tensor inversion based on the Wallace–Bott hypothesis^11,12^ provides rather loose constraints on the fit of a stress to a fault-slip datum^13^.

### Estimation of pore fluid pressure in the Philippine Sea slab

We next estimated the spatial distribution of pore fluid pressure in the Philippine Sea slab. The normal and shear stresses acting on a fault of any orientation (i.e., fault strike and dip-angle) within a stress regime are represented by Mohr circles^14^. The variation in the orientations of the focal mechanisms is attributed to fault strength heterogeneity caused by variation in the effective friction coefficient, *μ*′ which is represented by the ratio of normal stress to shear stress for each focal mechanism. In accordance with the principle of the law of effective stress, the effective friction coefficient *μ*′ can be defined as *μ*′ = *μ*(1 − *λ*)^15^, where *μ* is the friction coefficient and *λ* is the pore fluid pressure ratio. We classified the 36 earthquake events into three groups as F1, F2, and F3 to estimate *μ*′ for each fault group compatible only with the stress. Assuming a constant friction coefficient *μ* = 0.6^16^, the effective friction coefficients of groups F1, F2, and F3 were calculated as 0.02, 0.01, and 0.08, respectively (Fig. 2b). The coefficients show that the pore fluid pressure ratio *λ* is 0.85–0.97 in the Philippine Sea slab.

## Discussion

**Figure 3:**
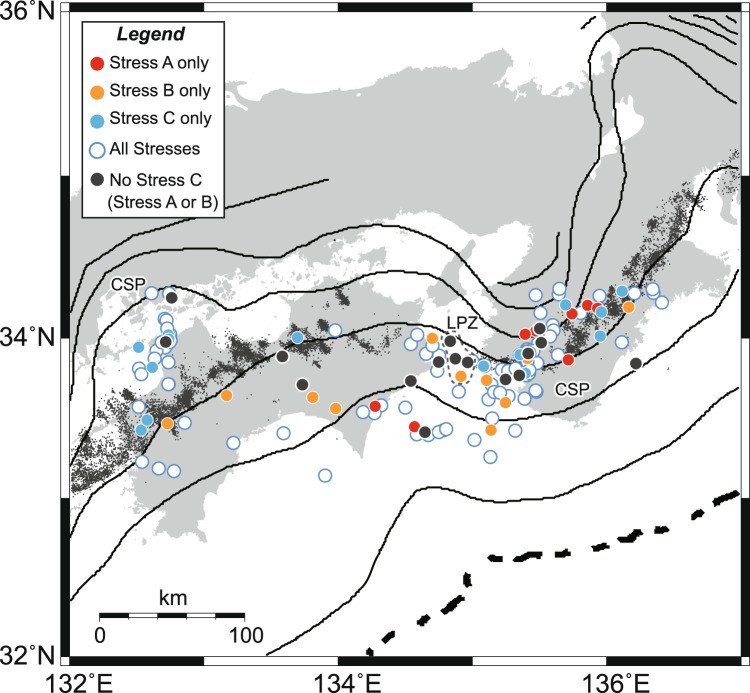
Spatial distributions of stresses determined using the multiple inverse method. The epicentres of the 164 seismic events examined are marked by colored circles. When a stress with a misfit of <30° is judged to be compatible with a fault-slip datum^8^, either or both of the stresses explain slip vectors on one of the nodal planes for 164 of the 188 events. Of the 164 events, only 36 are compatible only with a single principal stress, as the stress tensor inversion based on the Wallace–Bott hypothesis^11,12^ provides rather loose constraints on the fit of a stress to a fault-slip datum^13^. “All stresses” means that all the stresses can explain the slip vectors of the seismic events. “No Stress C” means that Stresses A and B can explain the slip vectors of the seismic events. Grey dots indicate the epicentres of nonvolcanic tremors in southwestern Japan for the period 2001 to 2009^32^. Depth contours indicate the upper surface of the Philippine Sea plate^33^. LPZ, Low permeable anisotropy zone (Gary dot region). CSP, Convex slab point.

Figure 3 shows spatial changes in the stress field obtained from the focal mechanisms for which observed slip directions are consistent with a single stress solution among the stresses. The focal mechanisms activated by Stress C dominate in the slab beneath the western and central regions of Shikoku, and the Kii Peninsula, whereas a wide region including the Kii channel includes focal mechanisms activated by Stresses A and B (Fig. 3). Beneath the Kii Peninsula and western and central parts of Shikoku, the gently dipping slab with a NW–SE strike has a convex-upwards shape (Fig. 1), as inferred from the distribution of intraslab earthquakes^17,18^ and from seismic tomography^19^. The intraslab earthquakes activated by the normal faulting stress (Stress C) are dominant in the region of convex-shaped slab (Convex slab point, CSP in Fig. 3). The resistance of the mantle to the margin-parallel component of oblique plate subduction controls the distribution of stress in the Philippine Sea slab^18^. Therefore, the heterogeneous stress distribution we obtained may be a function of the shape of the Philippine Sea slab. Future work will focus on spatial heterogeneities of stresses in the Philippine Sea slab by the detail seismicity study.

The results of our stress tensor inversion yield three major findings. First, the stress state in the Philippine Sea slab is spatially heterogeneous. Second, the triaxial normal faulting stress regime (Stress C) is confined to a limited part of the slab. Third, the pore fluid pressure ratio is high in the slab. We propose that the significant stress contrasts in the Philippine Sea slab cause regional variations in fluid migration within the slab. The permeability of rocks may be related to the σ_2_-axis direction^20,21^. True triaxial compression tests indicate that the σ_2_-axis direction is associated with the maximum permeability of rocks^22^. When the maximum permeability is dominant in the direction of σ_2_ and the hydraulic gradient has a component in the σ_2_ direction, fluids flow selectively in the σ_2_ direction^23^. Given this, we determined that Stress C, which has the intermediate stress ratio, gives rise to rocks with the greatest anisotropy of permeability. For Stresses A and B, there is a NNW–SSE-trending girdle of σ_1_-axes in a stereogram (Fig. 2a). The states of stress indicate a high stress ratio and thus σ_1_ ≈ σ_2_. Thus, the states of stress for Stresses A and B cause little anisotropy of permeability. The relationship between Stress C and hydromechanical properties indicates an anisotropy of permeability trending NNW–SSE subhorizontally in the slab beneath the Kii Peninsula and western and central regions of Shikoku.

The anisotropy of intraslab permeability causes regional variations in fluid migration in the Philippine Sea slab. This inference is consistent with the ^3^He/^4^He ratio distribution in the Kii Peninsula and western and central parts of Shikoku, which are regions of anomalous ^3^He/^4^He ratios. Recent studies^24–27^ have reported that the ^3^He/^4^He ratios in these regions show anomalously high values despite the fore-arc location, and the high ratios may be attributable to mantle helium derived from a magma source^28^. The inferred anisotropy of permeability in the region underlying the Kii Peninsula and western and central Shikoku is associated with high ^3^He/^4^He ratios. We are now able to explain, in terms of the heterogeneous distribution of stresses in the slab, why the region with a high ^3^He/^4^He ratios occur not only along the Kii Peninsula but also across the entire peninsula and western and central parts of Shikoku. Hence, the characteristic fluid migration mechanisms associated with Stress C are more favourable for generating fractures, which in turn facilitate the migration of mantle-derived helium.

**Figure 4:**
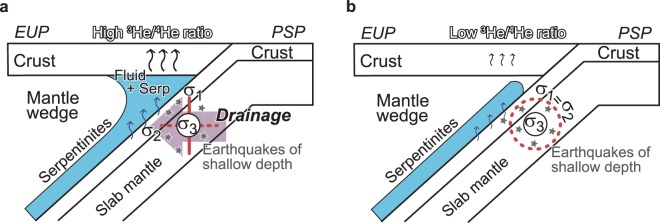
Schematic cross-section through the Nankai subduction zone. (**a**) Intraslab stress state of triaxial normal faulting stress with a NNW–SSE-trending subhorizontal orientation of the σ_2_-axis (Stress C). (**b**) Intraslab stress state (σ_1_ ≈ σ_2_) with a high stress ratio (Stresses A and B). The triaxial normal faulting stress promotes NNW–SSE-trending fluid drainage in the Philippine Sea slab. The accumulation of water and the build-up of pore fluid pressure in the corner of the mantle wedge, which are controlled by intraslab stresses, show along-slab heterogeneity. PSP, Philippine Sea Plate; EUP, Eurasian Plate.

The anisotropy of permeability caused by Stress C promotes fluid migration trending NNW–SSE subhorizontally. The high pore fluid pressure ratio (*λ* = 0.85–0.97) inferred by the present study is equivalent to a pore fluid pressure ratio *λ* = 0.95^29^ under the conditions of stress and temperature at depths where intraslab earthquakes occur. In the Nankai subduction zone, the fluids are liberated from the subducting slab at depth of 30–60 km^30^. This depth almost overlaps the depth of the earthquakes in the slab crust and mantle we used. If the spatial heterogeneity in fluid migration from the slab produces regional variations in the fluid volume, the accumulation of fluid and the build-up of high pore fluid pressure in the corner of the mantle wedge vary spatially along the slab. A relatively low value (*λ* = 0.85) of pore fluid pressure ratio in the slab with Stress C may support a large amount of dehydration to mantle wedge compared to the slab with Stresses A and B. We infer that slow earthquakes linked along dip in the Nankai subduction zone^4^ occur in the corner overlying the region of subducting plate where Stress C dominates. Stress C is not dominant in the Kii channel where slow earthquake activity is extremely low^4^ (Fig. 3). The region stretches ~10 km from ENE to SWS and ~20 km from NNW to SSE (See Low permeable anisotropy zone, LPZ in Fig. 3). In the region, the dehydration in Stresses A and B is not promoted only in a specific direction. The inhomogeneous conduit controlled by spatial heterogeneity in stress in the subducted Philippine Sea slab represents an intraslab fluid pathway, and is a plausible cause of the observed spatial heterogeneities in slow earthquake activity and ^3^He/^4^He ratio distribution (Fig. 4).

## Method

We applied the MIM^7^ to the data shown in Fig. 1. The MIM uses stress tensor inversions^15^ with a resampling technique for separating stresses from heterogeneous focal mechanism data^7^. Significant stresses are represented as clusters of reduced stress tensors and are identified by k-means clustering^31^.

## Data Availability

The datasets generated during the current study are available from the corresponding author on meaning request.

## References

[1] Terzaghi, K. & Peck, R. B. *Soil Mechanics in Engineering Practice* (Wiley, New York, 1967).

[2] Schmidt MW, Poll S (1998). Experimentally based water budgets for dehydrating slabs and consequences for arc magma generation. Earth Planet. Sci. Lett..

[3] Katayama I, Terada T, Okazaki K, Tanikawa W (2012). Episodic tremor and slow slip potentially linked to permeability contrasts at the Moho. Nature Geoscience.

[4] Obara K (2002). Nonvolcanic deep tremor associated with subduction in southwest Japan. Science.

[5] Matsubara M, Obara K, Kasahara K (2009). High-Vp/Vs zone accompanying non-volcanic tremors and slow-slip events beneath southwestern Japan. Tectonophysics.

[6] Mandl, G. *Faulting in Brittle Rocks: An Introduction to the Mechanics of Tectonic Faults* (Springer, Berlin, 2000).

[7] Otsubo M, Yamaji A, Kubo A (2008). Determination of stresses from heterogeneous focal mechanism data: An adaptation of the multiple inverse method. Tectonophysics.

[8] Nemcok M, Lisle RJ (1995). A stress inversion procedure for polyphase fault/slip data sets. J. Struct. Geol..

[9] Gephart JW, Forsyth DW (1984). An improved method for determining the regional stress tensor using earthquake focal mechanism data: application to the San Fernando Earthquake Sequence. J. Geophys. Res..

[10] Michael A (1991). Spatial variations in stress within the 1987 Whittier Narrows, California, aftershock sequence: new technique and results. J. Geophys. Res..

[11] Bott MHP (1959). The mechanics of oblique slip faulting. Geol. Mag..

[12] Wallace RE (1951). Geometry of shearing stress and relationship to faulting. J. Geol..

[13] Yamaji A, Otsubo M, Sato K (2006). Paleostress analysis using the Hough transform for separating stresses from heterogeneous fault-slip data. J. Struct. Geol..

[14] Jaeger, J. C. & Cook, N. G. W. *Fundamentals of Rock Mechanics, 3rd. edition* (Chapman and Hall, London, 1979).

[15] Angelier J (1989). From orientation to magnitudes in paleostress determinations using fault slip data. J. Struct. Geol..

[16] Byerlee J (1978). Friction of rocks. Pure and Applied Geophysics.

[17] Miyoshi T, Ishibashi K (2004). Geometry of the seismic Philippine Sea slab beneath the region from Ise Bay to western Shikoku, southwest Japan. Zisin.

[18] Wang K, Wada I, Ishikawa Y (2004). Stresses in the subducting slab beneath southwest Japan and relation with plate geometry, tectonic forces, slab dehydration, and damaging earthquakes. J. Geophys. Res..

[19] Nakajima J, Hasegawa A (2007). Tomographic evidence for the mantle upwelling beneath southwestern Japan and its implications for arc magmatism. Earth Planet. Sci. Lett..

[20] Sibson RH (1975). Generation of pseudotachylyte by ancient seismic pumping. Geophys. J. R. Astron. Soc..

[21] Sibson RH (2000). Fluid involvement in normal faulting. J. Geodyn..

[22] Takahashi M, Li X, Lin W, Narita T, Tomishima Y (2002). Permeability measurement techniques for intermediate principal stress direction. J. Japan Soc. Eng. Geol..

[23] Ogawa Y, Vrolijk P (2006). Control of internal structure and fluid-migration pathways within the Barbados Ridge decollement zone by strike-slip faulting: Evidence from coherence and three-dimensional seismic amplitude imaging: Discussion. Geol. Soc. Am. Bull..

[24] Umeda K, McCrank GF, Ninomiya A (2007). Helium isotopes as geochemical indicators of a serpentinized fore-arc mantle wedge. J. Geophys. Res..

[25] Sano Y, Nakajima J (2008). Geographical distributions of ^3^He/^4^He ratios and seismic tomography in Japan. Geochemi. J..

[26] McCrory PA, Constantz JE, Hunt AG, Blair JL (2016). Helium as a tracer for fluids released from Juan de Fuca lithosphere beneath the Cascadia forearc. Geochem. Geophys. Geosys..

[27] Wells RE, Blakely RJ, Wech AG, McCrory PA, Michael A (2017). Cascadia subduction tremor muted by crustal faults. Geology.

[28] Sano Y, Wakita H (1985). Geographical distribution of ^3^He/^4^He ratios in Japan: Implications for arc tectonics and incipient magmatism. J. Geophys. Res..

[29] Seno T, Yamasaki T (2003). Low-frequency tremors, intraslab and interplate earthquakes in Southwest Japan––from a viewpoint of slab dehydration. Geophys. Res. Lett..

[30] Yoshioka S, Toda M, Nakajima J (2008). Regionality of deep low-frequency earthquakes associated with subduction of the Philippine Sea plate along the Nankai Trough, southwest Japan. Earth Planet. Sci. Lett..

[31] Otsubo M, Sato K, Yamaji A (2006). Computerized identification of stress tensors determined from heterogeneous fault-slip data by combining the multiple inverse method and k-means clustering. J. Struct. Geol..

[32] Obara K, Tanaka S, Maeda T, Matsuzawa T (2010). Depth-dependent activity of non-volcanic tremor in southwest Japan. Geophys. Res. Lett..

[33] Hyndman RD, Wang K, Yamano M (1995). Thermal constraints on the seismogenic portion of the southwestern Japan subduction thrust. J. Geophys. Res..

[34] Seno T, Stein S, Gripp AE (1993). A model for the motion of the Philippine Sea Plate consistent with NUVEL-1 and geological data. J. Geophys. Res..

